# Characterizing alterations in attention networks under high mental workload

**DOI:** 10.1038/s41598-026-41477-4

**Published:** 2026-02-27

**Authors:** Lin Wu, Anping Ouyang, Xu Tang, Kewei Sun, Chaozong Ma, Tingwei Feng, Yijun Li, Lingling Wang, Huijie Lu, Xinxin Lin, Chenxi Li, Tian Zhang, Kuiliang Li, Peng Fang, Shengjun Wu, Daqing Huang, Lei Ren, Xufeng Liu

**Affiliations:** 1 Department of Military Medical Psychology, The Fourth Military Medical University, Xi’an, 710032 China; 2 School of Psychology, Shaanxi Normal University, Xi’an, 710062 China; 3 Teachers’ College, Beijing Union University, Beijing, 100011 China; 4 Military Psychology Section, Logistics University of PAP, Tianjin, 300309 China; 5 Military Mental Health Services & Research Center, Tianjin, 300309 China

**Keywords:** High Mental Workload, Attention Network, Behavioral Performance, Eye tracking, Visual Analogue Scale, Neuroscience, Psychology, Psychology

## Abstract

With the rapid development of modern society, occupational populations commonly face work states characterized by high mental workload (HMW). HMW can rapidly mobilize and deplete an individual’s cognitive resources within a short timeframe. As attention constitutes a fundamental cognitive quality, it is inevitably impacted by HMW, leading to issues such as impaired attentional function and decreased task performance. This study aimed to investigate the characteristics of alterations in attention network under HMW. Participants were recruited from a medical university using convenience sampling. The 1-back Stroop (BS) cognitive task was employed to induce an HMW state. The Attention Network Test-Revised (ANT-R) was completed before and after the induction of the HMW to assess attention network behavior. Eye tracking was assessed using an eye tracker, and subjective assessment was conducted using a visual analog scale (VAS). Statistical analysis was performed using a paired t-test or Wilcoxon signed-rank test. Subjective VAS ratings showed significant increases in mental fatigue (MF), mental effort (ME), mental stress (MS), boredom, and mind wandering (MW) following HMW induction compared to baseline. Behavioral results showed that, compared with before HMW state induction, there were significant differences in alert (sustained attention for maintaining arousal), moving+engaging (selective attention for adjusting and focusing on valid stimuli), flanker conflict (directional conflict between target stimuli and surrounding distracting stimuli) and location conflict (directional conflict between target stimuli and stimulus locations) changes. Alert efficiency values decreased significantly, while moving+engaging, flanker and location conflict values increased significantly. No significant differences were observed in the number of correct trials. Eye tracking results showed that, compared with the state before HMW induction, there were significant differences in average saccade duration and blink count, with a notable increase in both. The ANT-R demonstrates utility both as an experimental paradigm for assessing attention networks and as a behavioral indicator for evaluating HMW. Under the HMW state, significant changes were observed in a subset of behavioral, subjective rating scale, and eye-tracking indicators related to the attention network. This study validated the effectiveness of the BS cognitive task paradigm in inducing the HMW and provided new experimental evidence for revealing the characteristics of attention network changes in the HMW, laying the foundation for the development of assessment and intervention strategies.

## Introduction

 High Mental Workload (HMW) refers to a state characterized by insufficient capacity to cope with demands, arising from a relative deficiency in the cognitive resources required for complex information processing and cognitive operations during the execution of tasks with high cognitive demands^1,2^. In simpler terms, HMW represents an imbalance or mismatch between task demands and available cognitive resources. Research indicates that task demands primarily encompass two dimensions, task complexity and time pressure^3^. Task complexity involves the internal structure of the task, the amount of information involved, the degree of uncertainty, and the quantity of cognitive resources required. Time pressure denotes the time constraints for task completion and the resulting sense of urgency^4^. An individual’s cognitive resources encompass sustained attention, working memory, cognitive control, information processing abilities, etc^5^. High task demands, which simultaneously increase task complexity and time pressure, can rapidly mobilize an individual’s cognitive resources within a short period, leading to the intensive occupation and overload of their limited cognitive resources. When task duration is brief and mental workload is relatively low, cognitive resources are relatively abundant, enabling the individual to process task information effectively and maintain stable performance. However, as task duration extends, cognitive resources gradually diminish without timely replenishment. Although individuals may employ subjective effort to compensate and temporarily sustain task performance levels, this compensation cannot offset the rapid depletion of cognitive resources. Ultimately, this leads to performance decrements, potentially accompanied by issues such as increased mental stress, physiological and psychological fatigue, and reduced energy^6–9^.

HMW is typically caused by environmental factors, including high-pressure occupations, prolonged working hours, and work-related stress. In today’s society, many professionals (e.g., competitive athletes, military personnel, pilots, drivers, doctors, etc.) face work tasks that combine high task complexity with time pressure. They must maintain a high level of cognitive performance and respond quickly to unpredictable situations^10,11^. Prolonged exposure to HMW work environments not only adversely affects the physical and mental health of occupational populations but may also lead to reduced social productivity and innovation, trigger safety incidents, and result in severe consequences. For example, statistics show that approximately 10% to 40% of traffic accidents are caused by driver HMW or fatigue^12^; under HMW conditions, high-speed railway dispatchers exhibit significantly increased reaction times and reduced accuracy when handling complex tasks, which may be related to impaired prefrontal executive function^13^; HMW conditions negatively impact surgeons’ clinical performance, increasing error rates during patient treatment and leading to significant occupational burnout^14,15^. Therefore, research on HMW holds significant social importance.

Traditional HMW induction methods include laboratory cognitive tasks^16,17^, simulation tasks (e.g., simulated driving and simulated flying)^18,19^, and sleep deprivation, et al.^20,21^. Different types of methods have distinct induction characteristics, but they fundamentally remain closely aligned with task demands and time-on-task (ToT). Laboratory cognitive tasks are more commonly used due to their simplicity, controllable variables, and high reproducibility, and primarily include tasks such as the color-word Stroop task^22^, n-back task^23^, Time load Dual back (TloadDback) task^24^, Multi-attribute task battery-II (MATB-II)^25^, Toulouse n-back task (TNT)^26^, and Air traffic control (ATC) task^27^, et al. These tasks primarily manipulate task complexity (e.g., increasing the number of task components, information load, or uncertainty) and temporal pressure (e.g., shortening the response time window) to induce HMW. However, several existing tasks have notable limitations. For instance, single tasks such as the n-back or Stroop task have a relatively simple structure that only engages a limited range of cognitive abilities, lacking the concurrent assessment and recruitment of multiple cognitive functions, which results in insufficient intensity of HMW induction. In contrast, paradigms like MATB-II and ATC task involve high operational complexity and require specialized equipment and training, thereby restricting their widespread application. Additionally, simple and repetitive tasks may induce boredom and weariness in participants, which also leads to reduced task performance but is not attributable to an HMW state. The BS cognitive task is based on the cognitive resource depletion theory, integrating the color-word Stroop task and spatial 1-back task. It encompasses multiple cognitive domains including attention, working memory, inhibitory control, and cognitive flexibility, thus effectively enhancing task complexity. By setting appropriate response time limits for different task conditions, temporal pressure can be established; combined with the ToT effect, this paradigm enables rapid induction of the HMW state within a relatively short period. Previous studies have verified the efficacy of the BS cognitive task in HMW induction^28^. Its advantages of simple operation, controllable variables, and high reproducibility render it an ideal paradigm for HMW induction.

Recent studies have provided neurophysiological evidence and references for the evaluation of HMW. In terms of indicators, Dello Iacono et al. verified the sensitivity of the MDrow index derived from alpha-band activity by monitoring electroencephalogram (EEG) and other data during graded simulated driving tasks. This index has proven effective for mental fatigue assessment, with stability across both active and passive fatigue states as well as high and low cognitive load conditions^29^. A study on long-duration flight tasks demonstrated that EEG microstates are sensitive to mental fatigue, which plays a dominant role in modulating the dynamic changes of brain network microstates during simulated flight, suggesting that EEG microstates may serve as valid neural markers for monitoring large-scale brain network changes in real flight operations^30^. From the perspectives of cognitive resilience and resource compensation, Peng et al. found that enhanced variability in the frontoparietal network (FPN) interacting with alpha power can directly predict behavioral recovery during mental fatigue, providing potential indicators for evaluating recovery after mental fatigue intervention^31^. In terms of algorithms, researchers have proposed a feature extraction method combining mathematical morphology and the LSTM-CNN architecture, offering a feasible solution for establishing arbitrary-channel-based multi-factor EEG analysis in mental fatigue assessment^32^. Based on empirical evidence linking HRV to high mental workload or mental fatigue, many scholars have employed various data-driven machine learning algorithms, such as support vector machines (SVM) and K-nearest neighbors (KNN), achieving effective detection rates ranging from 75% to 91%^33,34^. In a flight scenario involving pilot-drone collaboration, significant differences have been observed in electrocardiogram and eye-tracking features under distinct cognitive load levels; electrocardiogram alone or combined with EEG monitoring yields favorable classification accuracy for HMW^35^. Regarding translational applications and devices, wearable equipment has been widely adopted for assessing HMW or mental fatigue, with continuous updates to meet higher demands of task and scenario adaptability^36,37^. Hamann et al. integrated existing assessment methods and empirical studies on mental fatigue, proposing a head-mounted sensing technique for evaluating mental fatigue in the cockpit and demonstrating its feasibility, thus promoting translational research from basic science to aviation applications^38^. Collectively, current research on the assessment of HMW and mental fatigue has achieved remarkable progress in the selection of sensitive indicators, the construction of intelligent algorithms, multimodal fusion, and the application of wearable devices. These studies not only provide reliable approaches for objective evaluation in laboratory settings but also gradually extend to real operational scenarios such as aviation and driving.

As one of the most fundamental cognitive abilities, attention ability is widely involved in emotion, decision, memory, and other psychological processes, thereby ensuring clearer comprehension, more accurate reactions, and more controllable behavior towards stimuli^39,40^. With deepening research into attention, psychologists such as Posner and Petersen have proposed the Attention Network Theory, based on studies of the brain structures and functions involved in attentional processes. This theory divides the attention network into three subnetworks: alert, orientation, and executive control^41^. The alert network refers to an individual’s ability to maintain a state of arousal, enabling effective perception of external stimulus information and reflecting the capacity for sustained attention^42^. Studies have demonstrated that the brainstem arousal system is critical for the maintenance of the alert state. In addition, structures such as the cerebral cortex (including the occipital, frontal, and parietal lobes) and subcortical regions (such as the superior colliculus and pulvinar nucleus of the thalamus) are also extensively involved in the process of alerting attention^43–45^. The orientation network refers to the ability of individuals to actively pursue task-relevant targets or passively follow suddenly appearing distractors, reflecting the capacity to selectively process external input information, known as selective attention^46^. Posner and Petersen (1990) proposed the role of the superior colliculus and pulvinar nucleus of the thalamus in attentional control and the allocation of attentional resources, a finding that has since been consistently verified by numerous subsequent studies^41^. It is now generally accepted that the process of orienting disengagement is primarily mediated by the parietal lobe, the midbrain circuit and superior colliculus are mainly involved in the process of orienting shifting, whereas the process of orienting engagement is predominantly regulated by the pulvinar nucleus of the thalamus^47^. The executive control network encompasses the monitoring and resolution of conflict. It participates in a series of processes, including planning, decision-making, error monitoring, responding to novel stimuli, and overcoming habitual behaviors, embodying the brain’s higher-level decision-making and active control abilities when facing conflict^48^. The brain regions activated by the executive control subnetwork include the bilateral prefrontal lobes, medial frontal areas, and anterior cingulate cortex, among other structures^49^. Although distinct functional subnets are each associated with their unique regulatory brain regions, it is noteworthy that the frontoparietal control network exhibits overlapping activation across the three subnetworks, a phenomenon that has been verified by some studies^50^. When an object of attentional focus enters the conscious state, it may activate the connections between the cingulate cortex and midline cortex, which reflects the alerting function subserving response anticipation^51^. In addition, the coordination between the cingulate cortex and lateral frontal regions is also involved in the executive control process of conflict resolution^52^. This may represent the physiological basis underlying the interactions among different subnets. Based on this, researchers have developed a series of Attention Network Tests (ANT) to assess and evaluate alert, orientation, and executive control functions simultaneously^53^. These include the original ANT, the child-friendly “fish” version ANT^54^, the dual-modality Attention Network Test-Interaction (ANT-I)^55^, the ANT-Revised (ANT-R) used to explore interactions between attention networks^38^, an auditory version for visually impaired populations, and versions used to investigate attention network changes under sleep deprivation and driving conditions^56,57^.

Among the methods for assessing attention networks, behavioral assessment is the most commonly used, providing core indices of attention network efficiency. By collecting reaction times (RT) and accuracy rates under various cueing conditions and conflict effects, the efficiency of the different subnetworks can be calculated. Since the ANT task requires participants to respond quickly and accurately to the direction of the target stimulus, this may lead to individual differences in the speed-accuracy trade-off^58,59^. Research indicates that when individuals are in a state of high alert, they often choose to sacrifice some accuracy for faster reactions to targets, resulting in decreased RT but increased error rates^41^. Although the three subnetworks represent distinct attentional qualities, they are not entirely independent of one another. For instance, a meta-analysis integrating data from 1,129 participants showed interactions between different cueing conditions and flanker conditions^60^. Therefore, there may be interactions between the alert and orientation networks obtained from different cue condition reaction time calculations and the executive control network efficiency obtained from flanker condition calculations. Research shows that higher alert in individuals can lead to greater flanker conflict, manifested as a decline in executive control function. Effective spatial cues can enhance executive control performance, while ineffective cues interfere with it^61^. Furthermore, different cue-stimulus onset asynchronies (SOA) can exert distinct effects: orientation exhibits a facilitation effect at shorter SOAs, where cued locations contribute to shortened reaction times; conversely, at longer SOAs, inhibition of return (IOR) occurs, resulting in delayed responses to target stimuli at cued locations. This phenomenon is considered to have a positive significance for improving attentional selection efficiency^62^.

Eye tracking is a technology capable of tracking eye tracking processes in real-time, accurately, and non-invasively. Different indices can be used to assess an individual’s cognitive processing characteristics, emotional states, and behavioral patterns^63,64^. For example, the distribution of fixation points and fixation durations can be used to evaluate an individual’s attentional focus and allocation of cognitive resources. Saccade amplitude and saccade velocity can be used to assess the efficiency and flexibility of attentional shifting. Studies have indicated that exogenous attentional shifting processes can be captured through saccadic indices, while endogenous attentional shifting may also involve the oculomotor system^65,66^. However, Remington et al. proposed that these two types of attention do not necessarily always accompany shifts in fixation^67^. Research by Schenk and Smith also demonstrated that endogenous attention can function through covert attentional processes without requiring overt eye tracking, independent of the oculomotor system^68,69^. The relationship between endogenous saccades and attentional shifting remains a topic of controversy. Some scholars argue that when the location of a target stimulus aligns with the direction prepared for an endogenous saccade, individual reactions are faster, suggesting that attentional shifting precedes endogenous saccades. In contrast, other scholars contend that endogenous saccades and attentional shifting are independent processes^70,71^. Yu et al. investigated the relationship between attention type and the accuracy of attentional shifting using endogenous and exogenous attention paradigms^72^. Results revealed that under valid cue conditions, both attention types significantly influenced saccade precision, with exogenous attention yielding higher accuracy in attentional shifting. Under invalid cue conditions, both types significantly affected saccade distance, with exogenous attention showing reduced accuracy in attentional shifting^72^. However, the assessment of attention networks using eye tracking indices is often influenced by individual cognitive processing strategies and eye tracking patterns, and their complex relationship requires further in-depth investigation.

This study employed the BS cognitive task to induce a HMW state. Behavioral methods, self-report scales, and eye-tracking techniques were used to investigate the characteristics of alterations in attention networks under HMW. This study provides novel experimental evidence for revealing the characteristics of attentional network changes under HMW states and lays the groundwork for developing assessment and intervention strategies.

## Methods

### Participants

Participants were recruited using campus posters. All participants were students from a medical university. Inclusion criteria were: (1) male; (2) aged 18–24 years; (3) normal or corrected-to-normal vision; (4) right-handed. Exclusion criteria were: (1) long-term unhealthy lifestyle habits (e.g., staying up late, smoking, or alcohol consumption); (2) use of medications or substances potentially affecting brain/autonomic nervous system function; (3) history of mental disorders or neurological diseases. A total of 98 participants were recruited for the study. Three participants withdrew during the experiment and did not complete the study. Data from three additional participants were excluded due to invalidity. Consequently, the final sample comprised 92 valid participants, all male, with a mean age of 21.06 ± 1.43 years (M ± SD). Prior to the study, all participants were informed about the relevant details, including the research purpose, procedures, privacy protection, voluntary participation, the right to withdraw, and the use of their data. All participants provided written informed consent indicating their understanding and agreement to participate. This study adhered to the principles of the Declaration of Helsinki and received approval from the Ethics Committee of the First Affiliated Hospital of the Fourth Military Medical University (No. KY20234188-1).

### Measurements

#### ANT

The ANT-R test, as reported by Fan et al. (2009)^48^, was used to concurrently evaluate the efficiency of the three attentional subnetworks: alert, orientation, and executive control. A central fixation cross (“+”) was displayed on the computer screen to focus participants’ fixation during intervals without cues or stimuli. Rectangular frames were presented horizontally on both sides of the fixation cross, serving to display cue and stimulus arrow combinations. Each trial consisted of two phases: the cue presentation phase and the stimulus response phase. Cues were categorized into four types: no cue, double cue, valid spatial cue, and invalid spatial cue. The stimulus arrow combination comprised five horizontally aligned arrows. The central arrow served as the target stimulus; participants were instructed to respond to its direction quickly and accurately by pressing a key. The four flanking arrows were identical in direction. The central arrow’s direction could be either congruent or incongruent with the flankers, creating the flanker conflict effect. The location of the stimulus arrow combination could be congruent or incongruent with the location of the preceding flashing cue, creating the location conflict effect. The efficiency of different attention sub-networks can be algebraically derived from reaction times (RTs) under different cue conditions and congruency conditions. For example: alert = RT_no cue_ − RT_double cue_^48^. Since its development, this test has been widely applied to measure attentional network efficiency. It holds significant value for investigating attentional development patterns in children and adolescents, selecting and evaluating special occupational populations (e.g., pilots, drivers), and assisting in the diagnosis and treatment evaluation of clinical conditions (e.g., Attention Deficit Hyperactivity Disorder, Alzheimer’s disease), demonstrating good reliability and ecological validity^73^.

#### HMW induction paradigm

The HMW state was induced using the BS cognitive task^28^. This task is grounded in the theory of cognitive resource depletion. It increases task demands by constructing both task complexity and time pressure, while simultaneously leveraging the ToT effect to induce the HMW state.

**Fig. 1:**
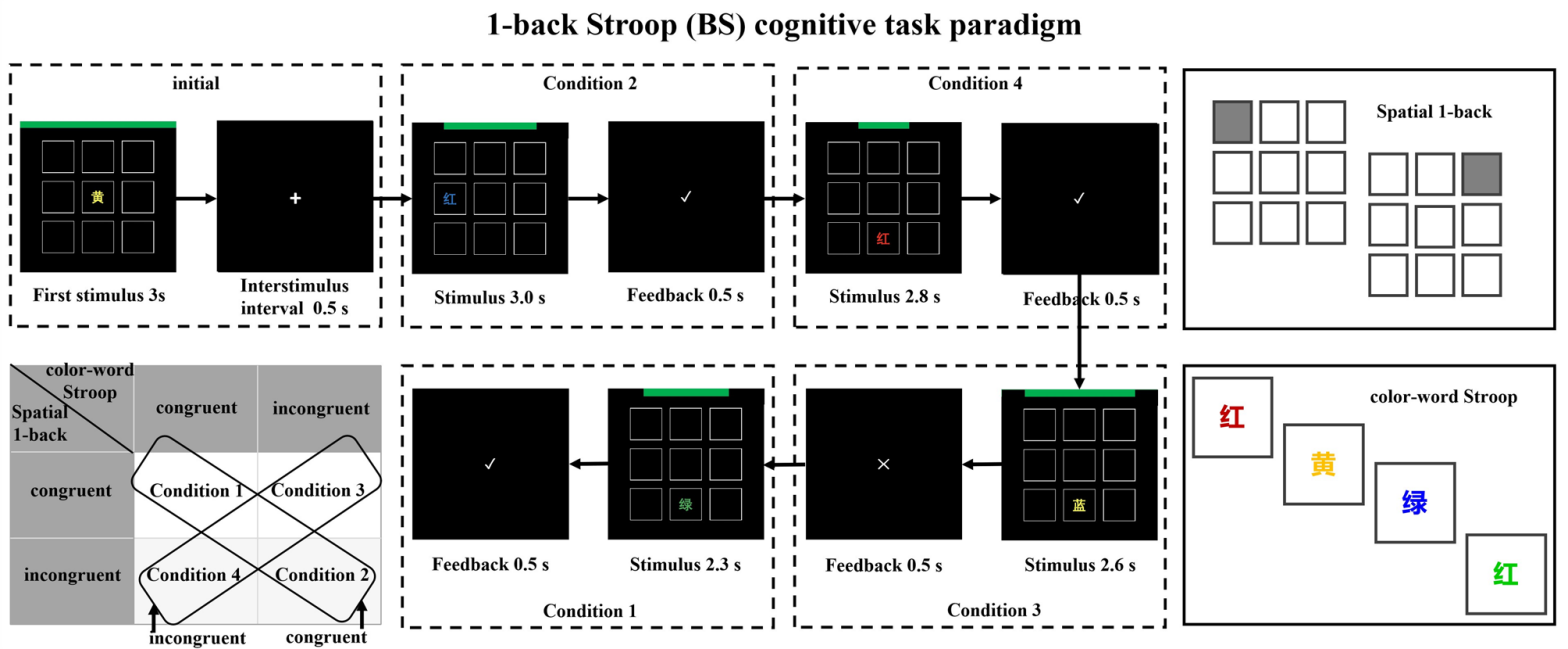
1-back Stroop (BS) cognitive task paradigm. 红 (Chinese) = Red (English); 黄 (Chinese) = Yellow (English); 蓝 (Chinese) = Blue (English); 绿 (Chinese) = Green (English).

Task complexity was constructed by combining the color-word Stroop task (a paradigm measuring inhibitory control ability) and the spatial 1-back task (a paradigm measuring working memory ability). Each trial presented one of the following four conditions: (1) Color-word Stroop task with identical color and meaning + spatial 1-back task with identical stimulus location; (2) Color-word Stroop task with different color and meaning + spatial 1-back task with different stimulus location; (3) Color-word Stroop task with different color and meaning + spatial 1-back task with identical stimulus location; (4) Color-word Stroop task with identical color and meaning + spatial 1-back task with different stimulus location. This task encompasses attention ability (participants must direct and concentrate their attention on multiple stimuli), working memory ability (in the spatial 1-back task, participants must store, compare, and update spatial location information of stimuli), inhibitory control ability (in the Stroop task, participants need to inhibit the dominant processing of word meanings and instead focus on the comparison of color and word meaning information), and cognitive flexibility (integrating and switching between the different conditions of the spatial 1-back task and the color-word Stroop task), totaling four cognitive abilities. The task has a high level of complexity. Time pressure can be controlled by the ratio of required time (RT) to available time (AT)^74^. Based on research exploration, the AT for the four conditions was set to 2300 ms, 3000 ms, 2600 ms, and 2800 ms, respectively^28^. The task duration is set to 60 min, consisting of six consecutive blocks, each lasting 10 min. Within each block, the four conditions of the BS cognitive task paradigm appear in a pseudo-random manner at a ratio of 1:1:1:1. Validated through self-report scales and behavioral experiments, this task effectively induces the HMW state. It also avoids the shortcomings of existing laboratory induction methods, which may cause low induction levels and prolonged emotional problems due to the simplicity of the task, resulting in poor results. This provides a new and effective method for effectively inducing the HMW state (Fig. 1).

#### VAS

The VAS was used to assess subjective psychological perceptions of attention networks under the HMW state. This scale quantifies an individual’s perceived intensity or experience of a specific stimulus or state^75^. The intensity level for each subjective feeling (i.e., item) is represented on a 10 cm straight line, increasing progressively from left to right. Participants were required to mark their current subjective experience level on this line. Researchers then quantified the score by measuring the distance in centimeters from the left endpoint to the mark. Scores ranged from 0 to 100, with a minimum unit of 1^76^. Based on previous literature, we included five items in this study: MF, ME, MS, boredom, and MW (e.g., “How did you feel mental fatigue during the previous task block?“). The meaning of each item was fully explained to participants before they took the measurement. Characterized by its simplicity, intuitiveness, rapid assessment capability, and high sensitivity, the VAS has been widely applied in fields such as medicine, psychology, and human-computer interaction, demonstrating good measurement efficacy^77^.

#### Eye tracking

The EyeLink 1000 Plus, developed by SR Research, is a high-precision eye tracker. It comprises a host computer for recording and analyzing eye tracking data, an infrared high-speed camera acquisition system, and accessories such as mounting brackets and chin rests. Infrared light rays that enter the eye are reflected by the cornea and retina and can be captured by a high-speed camera. Through high-frequency continuous sampling (up to 2000 Hz) and image processing algorithms, it effectively identifies and tracks the pupil center and corneal reflection center, thereby enabling rapid and accurate recording of participant eye characteristics, including pupil position, fixation points, and movement trajectories. This device features high precision and accuracy, high sampling rate and pupil resolution, and low-latency real-time tracking. The Experiment Builder (EB) software exhibits excellent compatibility with the EyeLink 1000 Plus eye tracker. This software enables the visual programming of eye-tracking tasks through drag-and-drop icons, supporting the triggering of various eye tracking events (e.g., fixations, velocity thresholds, saccades), which meets the requirements of diverse eye-tracking experimental paradigms. In this study, specific eye tracking measurement parameters include: (1) Sampling rate: 1000 Hz. (2) Mode: Single eye mode, stabilized head, 35 mm prism. (3) Distance (EyeLink 1000 Plus to eye): 60 cm. (4) Calibration procedure: 9-point calibration, with average error < 0.5°; recalibration was performed if the accuracy did not meet the requirement. (5) Saccade detection thresholds: Velocity threshold of 30°/s and acceleration threshold of 8000°/s². (6) Blink detection rules: Blinks were identified when the pupil area was less than 20% of the baseline pupil area for more than 10 ms. (7) Artifact handling: Data segments with excessive head movement (displacement > 2 cm) or calibration drift (error > 1°) were excluded. (8) Area of Interest (AOI) definition: AOIs included the central fixation cross, left and right rectangular frames, and stimulus arrow combinations within the frames. (9) Metric calculation: The eye tracking metrics reported in this study were calculated aggregated across the entire ANT-R task, reflecting global changes in visual attention state before and after the HMW induction. The experiment was conducted in the laboratory with consistent lighting intensity, and thick opaque curtains were closed throughout the entire experiment to eliminate the impact of lighting changes on eye-tracking indicators.

### Experimental procedure

**Fig. 2:**
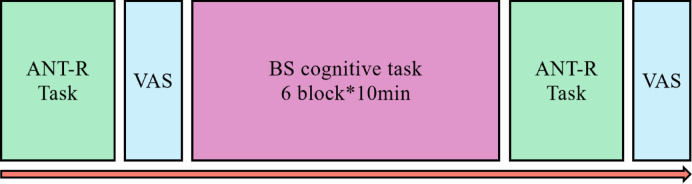
Experimental procedure. ANT-R: Attention Network Test-Revised; VAS: visual analog scale; BS cognitive task: 1-back Stroop cognitive task.

Behavioral and eye tracking assessments were conducted in the ANT-R task before and after the BS cognitive task. Scale assessments of the task state were conducted after the ANT-R task. The experimental procedure is illustrated in Fig. 2.

### Statistical analysis

Statistical analyses were performed using SPSS 25. Normality tests (Shapiro-Wilk test) were conducted separately on behavioral, scale, and eye tracking indices. If the normality assumption was met, paired-sample t-tests were used; if not, Wilcoxon signed-rank tests were employed. The significance level was set at α = 0.05.

## Results

### Subjective scales

**Fig. 3:**
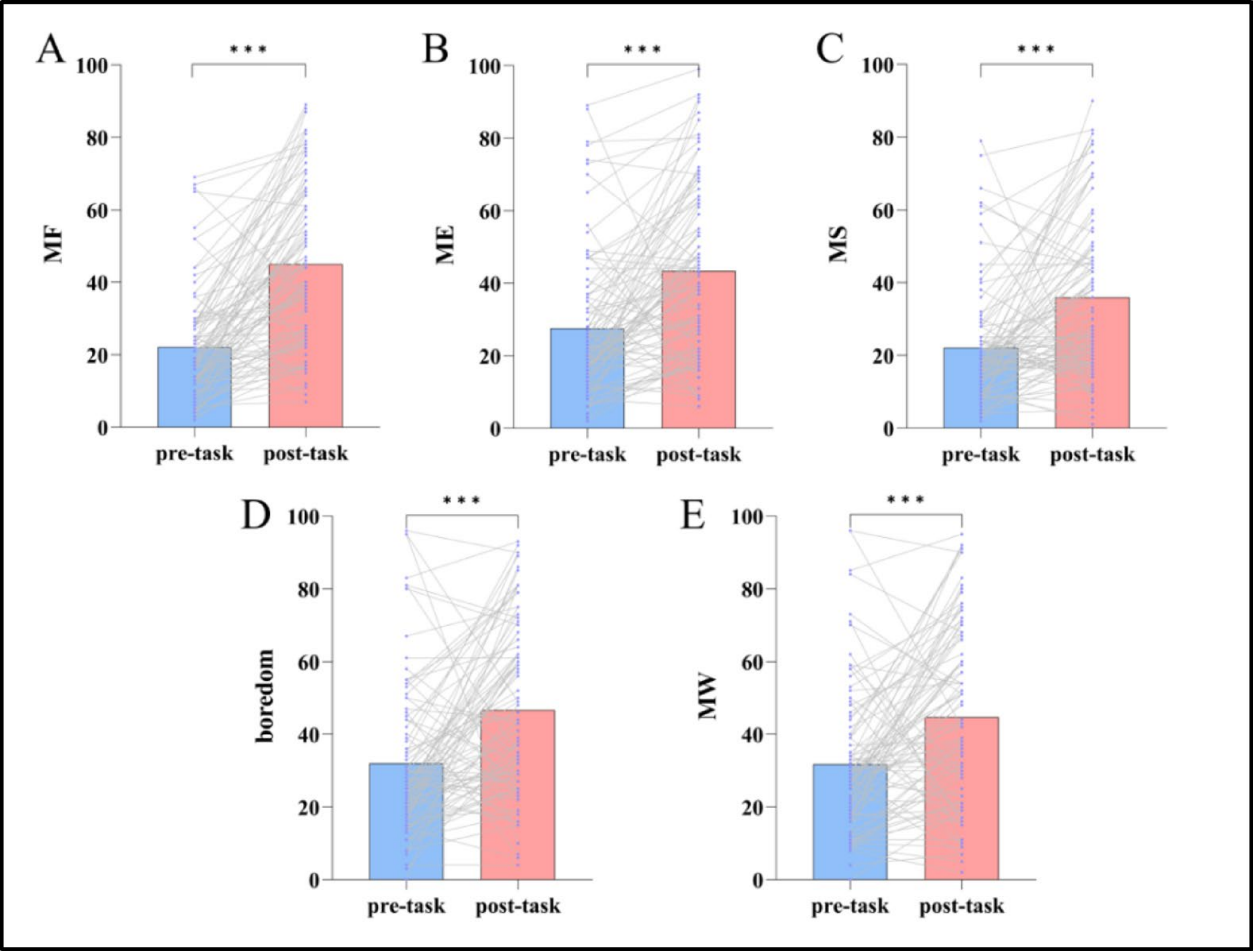
The results of VAS for the ANT-R before and after HMW induction. Purple dots represent participant data, and gray lines connect data from the same participant before and after HMW induction in the ANT-R task (*n* = 92). ^***^p < 0.001.

Normality test results for the VAS indicated that the differences in MF, ME, MS, boredom, and MW between the ANT-R tasks performed before and after HMW induction all satisfied the normality assumption (*Ps* > 0.05). Therefore, paired-sample t-tests were used for all indices. The results revealed significant differences in all indices before versus after HMW induction (*Ps* < 0.001) and all indices showed significant increases. See Table 1 Fig. 3.

**Table 1:** The results of VAS for the ANT-R before and after HMW induction ($\bar{X}$ ± s).

Index	Before induction	After induction	t	*P*
MF	22.12±14.98	44.98±22.13	-10.222	< 0.001
ME	27.54±19.79	43.36±23.57	-6.305	< 0.001
MS	22.10±15.95	35.96±21.90	-5.644	< 0.001
boredom	31.98±21.54	46.70±23.77	-5.103	< 0.001
MW	31.74±20.51	44.71±25.17	-4.704	< 0.001

### Behavioral performance

**Fig. 4:**
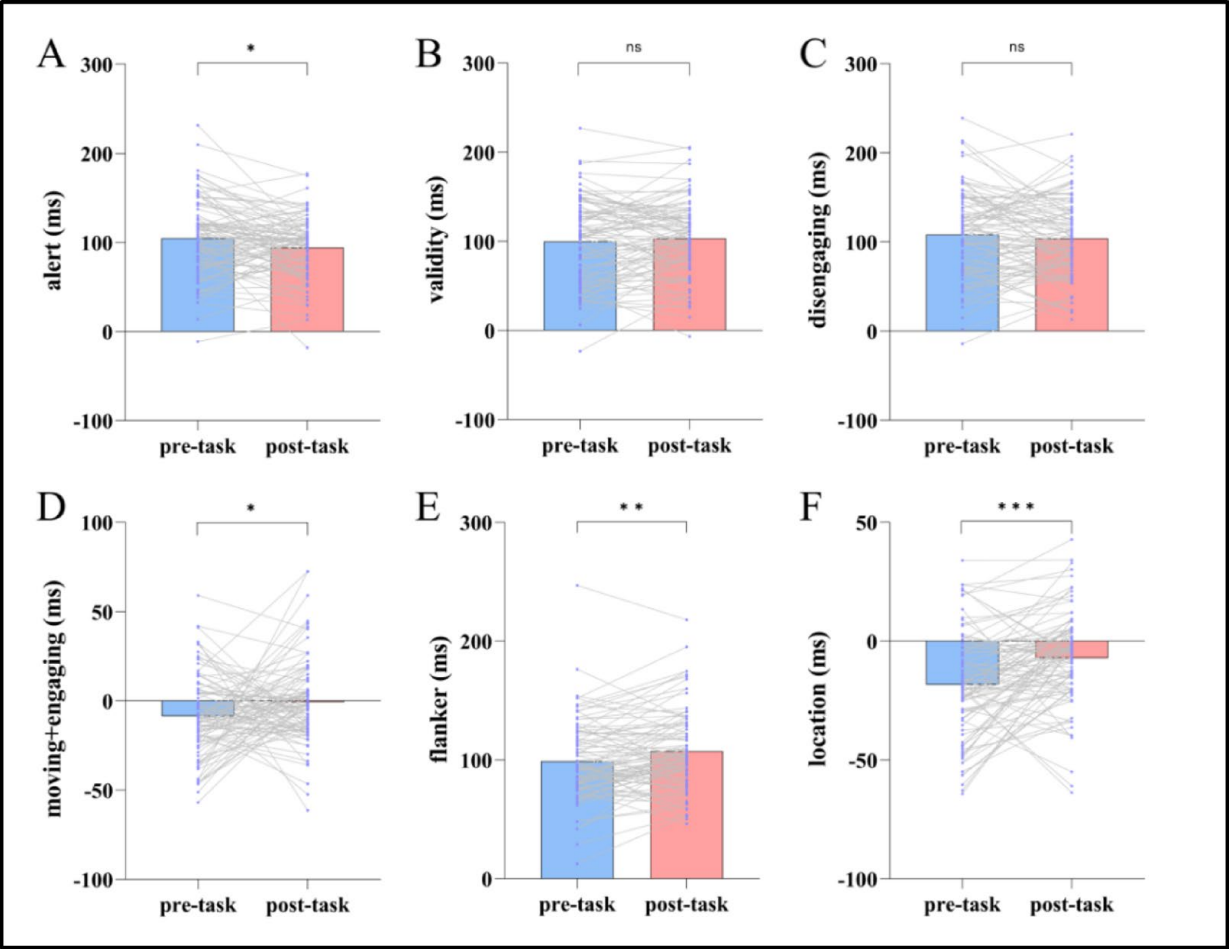
The results of ANT-R reaction time before and after HMW induction. Purple dots represent participant data, and gray lines connect data from the same participant before and after HMW induction in the ANT-R task (*n* = 92). ^*^p < 0.05, ^**^p < 0.01, ^***^p < 0.001.

Normality test results for RT indicated that the differences in alert, validity, disengaging, moving+engaging, flanker, and location between the ANT-R task performed before and after HMW induction all satisfied the normality assumption (*Ps* > 0.05). Consequently, paired-sample t-tests were used for all indices. The results revealed significant differences in alert, moving+engaging, as well as flanker and location conflict, before versus after HMW induction. Specifically, the alert value decreased significantly, while the values of moving+engaging, flanker and location conflict increased significantly. See Table 2; Fig. 4.

**Table 2:** The results of ANT-R reaction time before and after HMW induction ($\bar{X}$± s).

Index (ms)	Before induction	After induction	t	*P*
Alert	104.69 ± 43.00	94.21 ± 35.02	2.412	0.018
Validity	99.50 ± 47.19	103.04 ± 42.39	- 0.959	0.340
Disengaging	108.14 ± 48.87	103.93 ± 42.92	0.966	0.337
Moving+engaging	-8.64 ± 22.61	- 0.89 ± 23.38	- 2.156	0.034
Fanker	98.88 ± 33.84	107.43 ± 33.64	- 3.204	0.002
Location	-18.38 ± 21.98	-7.18 ± 19.73	- 4.551	< 0.001

Orientation includes disengagement (only in invalid cues), movement, and engaging.

Normality test results for correct trials indicated that the difference between the ANT-R tasks performed before and after HMW induction satisfied the normality assumption (*P* > 0.05). Therefore, paired-sample t-tests were used for the analysis of correct trials. The results showed that the count of correct trials was 123.88 ± 7.59 before HMW induction and 123.57 ± 7.86 after HMW induction. No significant difference was found in the correct trials before versus after HMW induction (t = 0.413, *P* = 0.681).

### Eye tracking

**Fig. 5:**
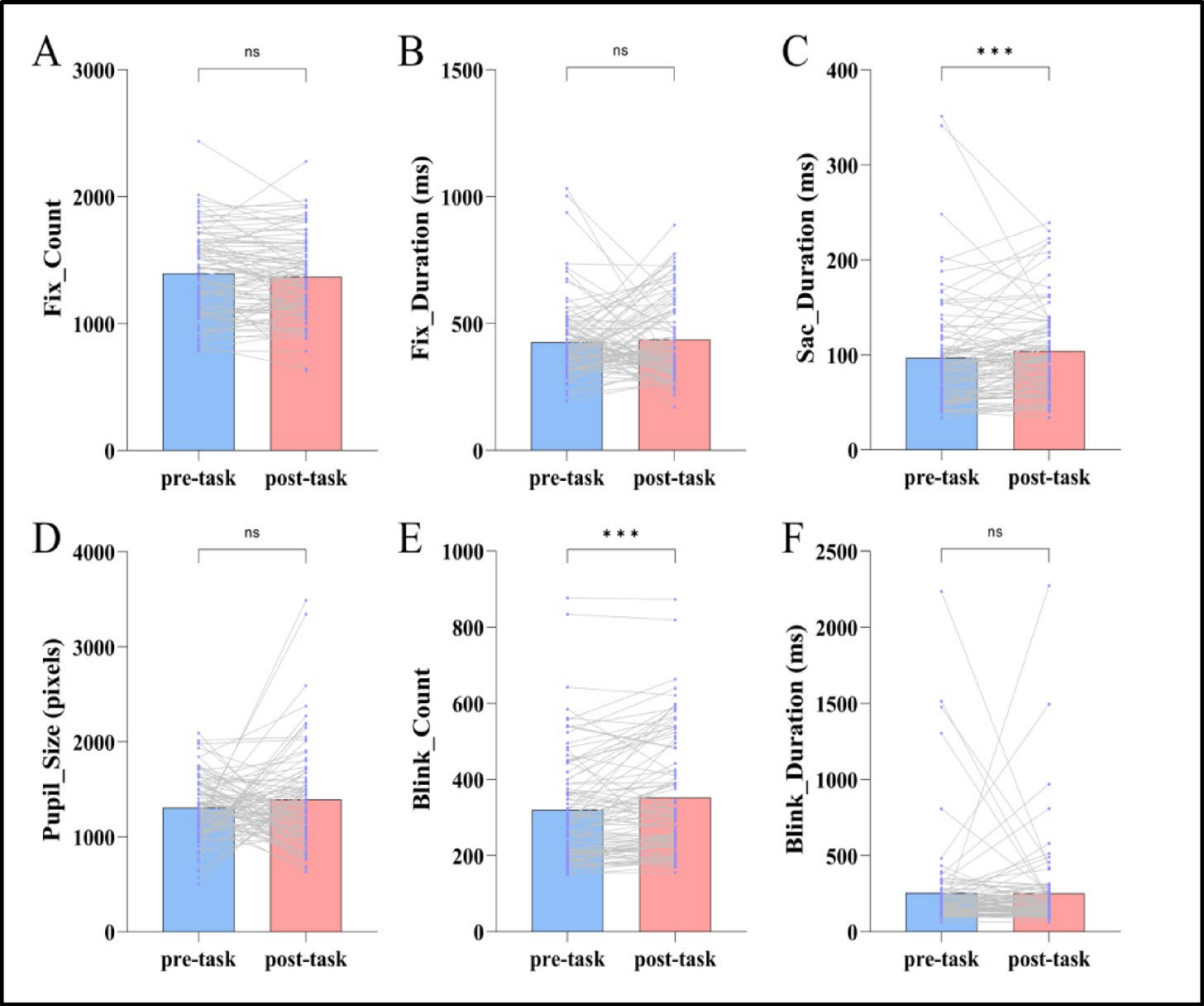
The results of eye tracking for the ANT-R before and after HMW induction. Purple dots represent participant data, and gray lines connect data from the same participant before and after HMW induction in the ANT-R task (*n* = 92). ^***^p < 0.001.

**Fig. 6:**
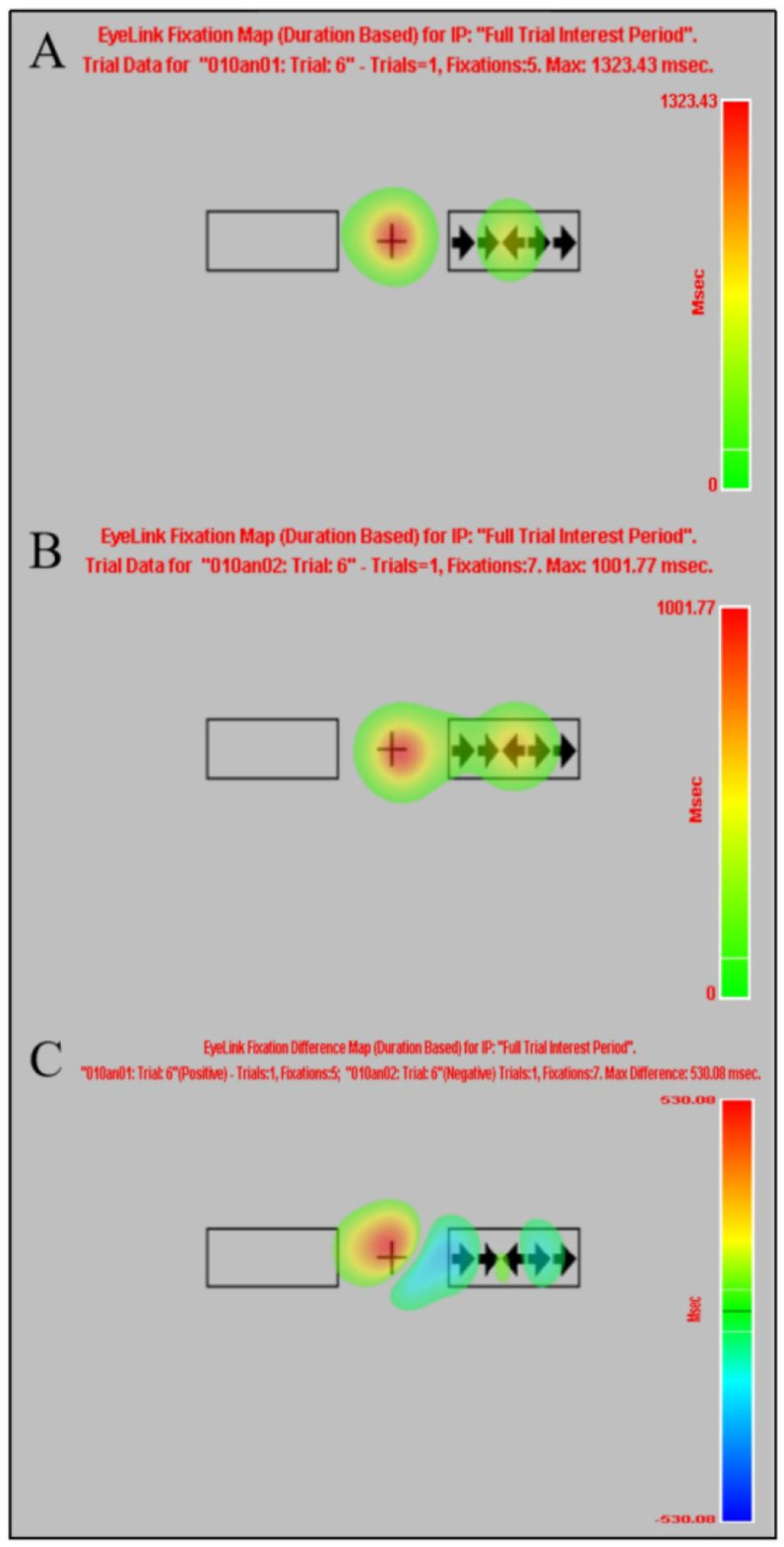
The fixation heatmap and fixation difference heatmap of the ANT-R task trial 6 for Subject 010 before and after HMW induction. (**A**): The fixation heatmap of the ANT-R task trial 6 for Subject 010 before HMW induction; (**B**): The fixation heatmap of the ANT-R task trial 6 for Subject 010 after HMW induction; (**C**): The fixation difference heatmap of the ANT-R task trial 6 for Subject 010 before and after HMW induction.

**Fig. 7:**
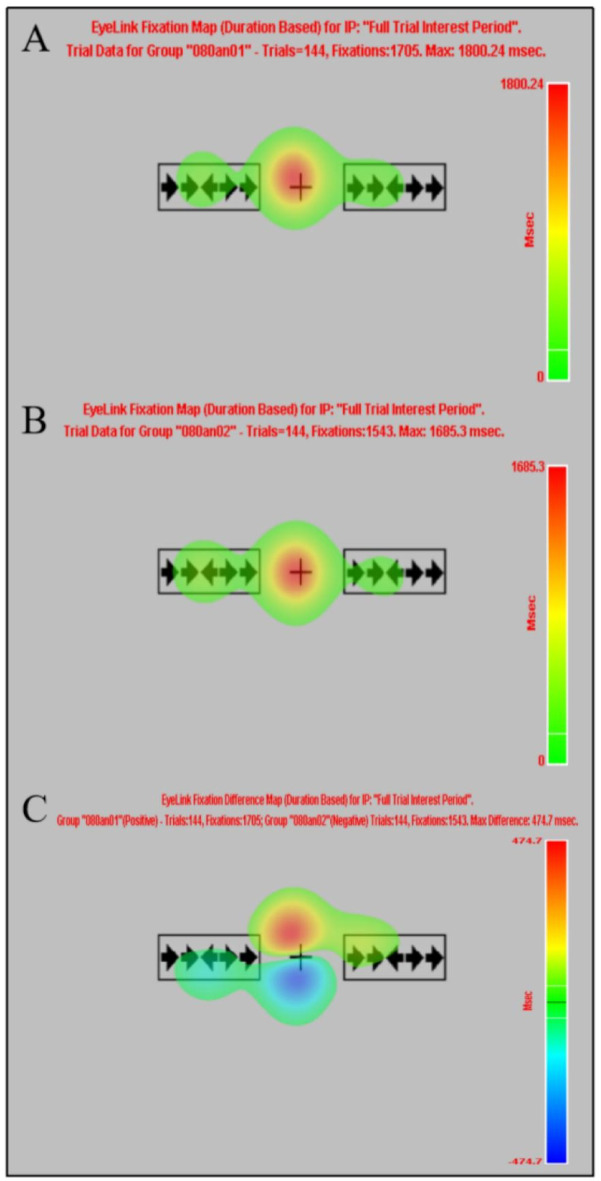
The fixation heatmap and fixation difference heatmap of the ANT-R task for Subject 080 before and after HMW induction. (**A**): The fixation heatmap of the ANT-R task for Subject 080 before HMW induction; (**B**): The fixation heatmap of the ANT-R task for Subject 080 after HMW induction; (**C**): The fixation difference heatmap of the ANT-R task for Subject 080 before and after HMW induction.

Normality test results for the eye tracking indicated that the Fix_Duration difference in the ANT-R before and after HMW induction satisfied the normality assumption (*P* > 0.05), while the Fix_Count, Sac_Duration, Pupil_Size, Blink_Count, and Blink_Duration differences did not satisfy the normality assumption (*Ps* < 0.05). Therefore, paired-sample t-tests were used for Fix_Duration, while Wilcoxon signed-rank tests were employed for the remaining indices. The results revealed significant differences in Sac_Duration and Blink_Count before versus after HMW induction. Specifically, Sac_Duration was significantly prolonged, and Blink_Count significantly increased. See Table 3; Fig. 5. The fixation heatmap and fixation difference heatmap of the ANT-R task trial 6 for Subject 010, both before and after HMW induction, are shown in Fig. 6; the fixation heatmap and fixation difference heatmap of the ANT-R task for Subject 080, both before and after HMW induction, are presented in Fig. 7 (the results presented in fixation heatmaps Figs. 5 and 6 are reported solely for illustrative purposes, and the findings at the trial level and individual level should not be overinterpreted as conclusive evidence).

**Table 3:** The results of eye tracking for the ANT-R before and after HMW induction ($\bar{X}$± s).

Index	Before induction	After induction	z/t	*P*
Fix_index				
Fix_count	1393.85±335.42	1366.97±320.99	- 1.040	0.298
Fix_duration (ms)	426.38±155.03	436.83±157.88	- 0.470	0.639
Sac__index				
Sac_duration (ms)	96.71±56.40	104.02±46.69	- 3.310	＜0.001
Pupil_index				
Pupil_Size (pixels)	1303.56±340.71	1389.48±515.65	- 0.522	0.602
Blink_index				
Blink_count	319.93±143.26	352.28±156.33	- 3.559	＜0.001
Blink_duration (ms)	255.18±321.89	252.41±288.48	- 0.892	0.373

## Discussion

### Behavioral performance

Behavioral indices included alert, orientation (validity, disengaging, and moving+engaging), and executive control (flanker conflict and location conflict). Alert is the ability to respond quickly to stimuli and maintain a state of readiness, i.e., the ability to detect stimuli; orientation is the ability to select and track targets, i.e., the ability to select stimuli; executive control is the process of making high-level decisions and actively controlling stimuli, i.e., the ability to process stimuli^78^. The results of this study indicate that after HMW induction, the alert subnetwork efficiency significantly decreased. Since the efficiency of the alert subnetwork is reflected by the difference between the average RT under the no-cue condition and the average RT with double clues^79^, the significant decrease in post-induction values indicates that the RT with double clues after induction is significantly longer than that before induction, meaning that the response to stimuli after the cue appears is slower, resulting in a significant decline in the performance of the alert subnetwork. For the moving + engaging, the significant post-induction increase in values indicates that the RT to double cues was significantly longer (with larger values) after induction than before. This suggests that the orientation efficiency for ambiguous cues (double cues) was reduced. For flanker conflict and location conflict, the significant post-induction increase in values indicates that the RT to incongruent stimuli was significantly longer (with larger values) after induction than before. Specifically, participants’ responses to incongruent information were significantly slowed down post-induction, reflecting a decline in executive control efficiency. These results suggest that the detrimental effects of the HMW state on attention ability encompass all subnetworks, findings that have been confirmed by relevant studies^80,81^.

Behavioral indices can be evaluated based on data regarding the actual task completion performance, directly and authentically reflecting task performance. They are thus regarded as core indicators for performance assessment^82–84^. During task execution under HMW, different modal indices for an individual may exhibit varying changes. For instance, subjective fatigue may intensify with prolonged task duration, blink frequency may increase due to ocular fatigue, and heart rate may accelerate in response to heightened stress. However, if an individual’s behavioral performance remains at a good level without affecting the effective completion of tasks, then the individual is still competent for the task in HMW state. Conversely, if an individual’s behavioral performance declines, rendering them unable to complete the task accurately and effectively, they are incompetent for the task under that state, even if other modal indices (e.g., scales, eye tracking, EEG, heart rate variability) show no significant changes. Numerous studies have demonstrated that the HMW state leads to reduced attentional task performance, which may or may not be accompanied by changes in multi-modal indices^80,81^. A study inducing HMW using a 60-minute continuous arithmetic task reported no decline in behavioral efficiency of attention network. Instead, it observed reduced resources for early-stage stimulus processing in the alert network, manifested as decreased N1 amplitude in EEG; reduced resources for the categorization process in the orientation network, manifested as decreased P3 amplitude; and reduced resources for response conflict and inhibition in the executive control network, manifested as decreased N2 and P3 amplitudes^85^. These findings also corroborate the potential asynchrony between behavioral indices of attentional networks and results from other modalities. Divergent results may be attributable to differences in the degree of HMW induction, individual motivation and effort levels, and the meaning and sensitivity of different modal indices, warranting further in-depth investigation in future studies.

In this study, the ANT-R task serves a dual purpose: it functions both as an experimental paradigm for assessing attention networks and as a behavioral measure for evaluating the HMW state. Under the HMW state, the performance of the alert, orientation, and executive control subnetworks of the attention network is significantly reduced. This not only validates the effectiveness of the BS cognitive task paradigm in inducing HMW state but also reflects the characteristics of HMW state’s impact on the attention network, providing a new and effective method for inducing HMW state and studying its effects.

### Subjective scales

Subjective scale indices included MF, ME, MS, boredom, and MW. As their names suggest, MF represents the level of fatigue induced by the task, ME represents the degree of effort expended to complete the task effectively, MS represents the level of stress, boredom is the emotional experience during the task, and MW is a process involving the unconscious, automatic shifting of thoughts. The results of this study indicate that all VAS scores increased significantly, and subjective experiences worsened significantly.

MF serves as the most direct subjective assessment of the HMW state. The significant increase in MF scores demonstrates that participants were indeed in an HMW state. Many research indicates that MF intensifies with prolonged task duration, regardless of task difficulty, reflecting the cumulative ToT effect^85^. In the present study, participants consecutively performed the pre-HMW induction ANT-R, the BS cognitive task paradigm for HMW induction, and the post-HMW induction ANT-R. The entire procedure lasted approximately 90 min without breaks, concurrently engaging complex cognitive abilities including attention and its subnetworks, working memory, inhibitory control, and cognitive flexibility. In this state, it is understandable that cognitive resources are heavily depleted and participants experience high levels of MF.

ME is considered to be generated by individuals relying on non-automated cognitive control processes when performing cognitive tasks^86^. Moderate ME is positively correlated with task performance; higher goal-directedness and achievement motivation prompt individuals to more actively mobilize cognitive resources and employ cognitive strategies, facilitating better task completion and enhancing positive psychological qualities such as self-esteem and self-efficacy. However, excessive MF may conversely accelerate the depletion of cognitive resources, leading to diminished task performance^87,88^. During the post-HMW induction ANT-R task, participants exhibited a broad decline in overall attention network behavioral efficiency. Crucially, despite subjectively investing greater ME, this increased effort was insufficient to compensate for the decline in efficiency. This suggests that the HMW state had a detrimental effect on participants, both subjectively and objectively^89^.

MS represents the level of stress that individuals subjectively experience during the task. It is generally accepted that an inverted U-shaped relationship exists between MS and task performance. Within a certain range, moderate MS prompts individuals to invest greater ME. However, beyond a specific threshold, excessive MF can instead lead to diminished task performance and adversely affect mental health. Participants reported greater MS after HMW induction, consistent with the findings of Szalma et al.^90^.

Boredom refers to an affective experience characterized by feelings of weariness, loss of interest, or lack of vitality in a specific environment or task^91^. It is generally believed that individuals are less likely to report high levels of boredom during cognitively demanding tasks. Conversely, higher levels of boredom may be reported during tasks that are relatively simple, less time-constrained, and monotonously repetitive^92^. Compared to the BS task paradigm, characterized by high difficulty and time pressure, the ANT-R task only required key-press responses to the direction of the central arrow. Spanning 144 trials, this task might potentially be perceived as a “simple and repetitive” task by university students, possibly contributing to the significant increase in boredom.

MW denotes the process whereby an individual’s attention involuntarily drifts away from the current task-related activities towards task-unrelated thoughts^93,94^. Killingsworth et al. from Harvard University surveyed 2,250 participants and found that the proportion of individuals experiencing MW during wakefulness was as high as 46.9%, suggesting that attention is not consistently focused on the immediate environment or tasks^95^. Scholars Smallwood and Schooler proposed the executive control theory of MW in 2006, which states that MW occurs when an individual’s executive control system fails to control non-task-related information that intrudes into their thoughts, thereby elevating the occurrence and development of MW to a higher level of cognitive control^96^. In the present study, the HMW state consumed substantial cognitive resources, which could not be rapidly replenished due to the continuous demands of the task. This resulted in a persistent decline in executive control resources, rendering them ineffective at shielding against the intrusion of task-unrelated thoughts, ultimately leading to a significant increase in MW.

In summary, the significant changes in MF, ME, MS, boredom, and MW subjectively validated the effectiveness of the HMW state induction, indicating the experience and perception of the adverse effects of the HMW state on attention network performance.

### Eye tracking

Eye tracking indices reflect the attention process by directly providing characteristics of where, how long, and how an individual looks at something during a task^97^. Diverse eye tracking indices exist, and research indicates that fixation indices, saccade indices, pupil indices, and blink indices each possess distinct advantages in revealing mental workload and attentional capacity^98–101^. This study included six indices, namely Fix_Count, Fix_Duration, Sac_Duration, Pupil_Size, Blink_Count and Blink_Duration, to assess attention network function under HMW state.

Fix_Count refers to the number of times the eyes focus on a target within an area of interest, and is one of the most basic indices of eye tracking. It reflects an individual’s level of interest in a particular location and involves the perception and processing of stimulus-related information^102^. Fix_Duration refers to the time interval from when the eyes move to a fixation point until they depart from that point, i.e., the time spent at the fixation point^102^. Research indicates that under HMW state, Fix_Count and Fix_Duration exhibit an increasing trend. This is because, at this time, the individual’s cognitive resources are highly depleted, leading to reduced efficiency in target search, encoding, and feedback, which necessitates more fixation points and longer fixation duration to adequately process information^103,104^. In this study, although Fix_Count showed a decreasing trend and Fix_Duration showed an extending trend, they had not yet reached a significant difference. This may be influenced by various factors such as task complexity, HMW severity, attention strategies, and individual differences in tolerance to HMW. For example, in low-complexity tasks and familiar tasks, individuals may engage in more automated processing while reducing fixation count and duration on unnecessary information to concentrate cognitive resources on deeply processing effective information.

Sac_Duration refers to the duration of eye movements between a series of fixation points^105^. Whether it is top-down, goal-driven voluntary saccades or bottom-up, stimulus-driven reflexive saccades, Sac_Duration is an important indicator for measuring the attention ability to quickly orient and capture stimuli^106^. Tokuda et al.‘s research suggests that saccades may be a more accurate indicator of HMW than pupil diameter^107^. This study found that Sac_Duration was significantly prolonged in the ANT-R task after HMW induction, indicating a reduction in the orientation efficiency, which required more time to effectively capture stimuli, consistent with the conclusions of existing studies.

The pupil is the circular opening at the center of the iris, regulated by the balance between the sympathetic and parasympathetic nervous systems^108^. The pupil is not only a crucial structure for light regulation in the visual system but is also closely associated with factors such as cognitive load, emotional state, and arousal level^109–111^. Hess et al. first proposed using pupil size as an indicator of cognitive load in the 1960s, demonstrating a positive correlation between pupil diameter and task difficulty^112^. Their findings were published in *Science*, marking the beginning of a series of studies on the relationship between pupil size and cognitive load^113^. Task-evoked pupillary responses (TEPR) have been shown to increase pupil dilation with increasing cognitive and mental workload, not only in visual tasks, but also in listening tasks. Pupil dilation induced in speech processing tasks can be interpreted as the result of processing load and cognitive effort^114–116^, indicating that pupil changes triggered by HMW tasks are consistent across perceptual channels. Although no significant difference in Pupil_Size was found before and after HMW induction in this study, the Pupil_Size still showed an increasing trend after induction, which was basically consistent with the existing conclusions.

Blinking, the rapid closing and opening of the eyelids, has been demonstrated to be a sensitive indicator reflecting HMW states and sustained attention^117,118^. During periods of focused attention, individuals exhibit relatively fewer blinks to ensure rapid and effective responses to external stimuli. Under sustained HMW, Blink_Count tends to increase. Zargari Marandi et al. interpret this as the development of mental workload leading to the deactivation of blink inhibition^119^. We posit that this may indicate impaired alert, making it difficult to sustain high-intensity attentional resource allocation, while individuals also attempt to alleviate ocular fatigue and restore attentional capacity through blinking. However, frequent blinking may delay or cause the omission of perception of continuous stimuli, resulting in diminished task performance. Regarding the Blink_Duration, there is a view that under fatigue conditions, this indicator may be prolonged due to weakened contractions of the levator palpebrae superioris muscle^120^. This study found that Blink_Count significantly increased after HMW induction, consistent with the above conclusion; Blink_Duration remained largely unchanged compared to pre-induction levels, showing no significant difference. This suggests that individuals did not employ a strategy involving prolonged eye closure for rest regulation during the task.

Heatmaps are used to display the spatial distribution of fixation points on stimulus materials, reflecting an individual’s attention allocation patterns through eye tracking characteristics. They offer excellent visualization effects and have become an important tool for studying visual attention and cognitive processing^121^. Red or yellow areas indicate higher fixation point density, longer processing times, and deeper processing; blue or green areas indicate lower fixation point density, shorter processing times, and shallower processing. Trial heatmaps and difference heatmaps are illustrated using Trial 6 of the ANT-R task before and after the HMW for subject 010 (the same cue-stimulus pattern ensures comparability). Before the cue and stimulus appeared, participants’ fixation points were concentrated around the “+”; when the stimulus appeared, fixation points and dwell time were predominantly concentrated on the central arrow, indicating that the stimulus effectively directed participants’ attention and that participants adhered well to the task rules. After HMW induction, the duration of the participants’ fixation on the central arrow was longer than that before induction, indicating a longer processing time; simultaneously, fixation points were more widely distributed on the right side, suggesting that participants may have failed to directly and effectively locate the central arrow or conducted directional comparisons between the central and surrounding arrows to confirm the correct response, resulting in reduced orientation and executive control efficiency. The difference heatmap also validated the above results. These findings align with the characteristics of HMW state effects on cognitive and attention functions. Task heatmap and difference heatmap using subject 080 as an example, before and after HMW induction in the ANT-R task (the arrow combinations on both sides of the frame are for illustrative purposes only). Similarly, the stimulus triggered the participants’ effective attention orientation, and the participants also followed the task rules relatively well. While the overall fixation patterns before and after HMW induction were largely consistent, the difference heatmap revealed a disparity in fixation points and duration on the central fixation cross (“+”). After the HMW induction, the participant’s fixation points were positioned lower, suggesting that factors such as muscular fatigue, attentional resource reallocation, and an altered emotional state under HMW may have contributed to a downward fixation shift. It should be reiterated that the fixation heatmaps Figs. 6 and 7 are provided for illustrative purposes only, and the findings at the trial level and individual level should not be overinterpreted as conclusive evidence.

### Limitations

Several limitations of this study should be acknowledged. First, limitations in participant sampling. Participants were exclusively male students recruited from a medical university, limiting their representativeness of all occupational and demographic groups. The influence of different demographic factors may vary. Additionally, the use of convenience sampling restricts the generalizability of the findings. Future research should incorporate participants of diverse ages, genders, and occupations, employing more scientifically rigorous sampling methods to enhance the ecological validity of the results. Second, limitations in study design. The simple pre-test–post-test design has limitations in establishing a rigorous causal relationship. In future research, we will adopt methods such as a control group design and repeated measures to control for extraneous factors, thereby further clarifying the causal relationship between HMW and attention network impairment. Third, limitations in task design. The BS cognitive task paradigm used for HMW induction in this study remains a traditional laboratory-based behavioral task, which may not fully replicate HMW states in real-world operational environments. Future studies could integrate technologies such as virtual reality (VR), incorporate multi-channel task stimuli, and optimize cognitive tasks to better approximate real-world work scenarios. Fourth, limitations in research methodology. This study used behavior, scales, and eye tracking methods to investigate changes in the attention network under HMW state, but failed to delve into the neural mechanism level, and there was a lack of integration between indices. Future research could supplement with electroencephalography and utilize data fusion algorithms (e.g., machine learning) to explore the deeper relationships between different indices further.

## Conclusions

This study employed the BS cognitive task paradigm to induce an HMW state. The ANT-R task was administered both before and after HMW induction. Behavioral indices, self-report scales, and eye tracking indices collectively revealed characteristic alterations in attention subnetworks. Under the HMW state, the efficiency of the alert, orientation, and executive control subnetworks significantly decreased, and MF, ME, MS, boredom, MW as well as saccade duration and blink count significantly increased. This study validated the effectiveness of the BS cognitive task paradigm in inducing the HMW state, and provided new experimental evidence for revealing the characteristics of attention network changes under the HMW state, and laid the foundation for the development of assessment and intervention strategies.

## Data Availability

The data of this study are available from the corresponding author upon reasonable request.
